# Consensus for criteria of running a pediatric inflammatory bowel disease center using a modified Delphi approach

**DOI:** 10.1007/s12519-023-00691-0

**Published:** 2023-04-06

**Authors:** You-You Luo, Kai-Chun Wu, Si-Tang Gong, Ying Huang, Hong Yang, Qing-Ya Tang, Ying-kit Leung, Jie Wu, Lan-Lan Geng, Wei Zhou, Mei Sun, Chao-Min Wan, Zai-Ling Li, Ying Fang, Xiao-Qin Li, Mei Li, Zhao-Xia Wang, Yuan Xiao, Xue-Mei Zhong, Xiao-Fei Chen, Jie Chen

**Affiliations:** 1 Department of Gastroenterology, Children’s Hospital, Zhejiang University School of Medicine, Hangzhou, 310052 China; 2 National Clinical Research Center for Child Health, Hangzhou, 310051 China; 3 Department of Gastroenterology, Xijing Hospital, Fourth Military Medical University, Xi’an, 710032 China; 4 Department of Gastroenterology, Guangzhou Women and Children’s Medical Center of Guangzhou Medical University, Guangzhou, 510623 China; 5 Department of Gastroenterology, Children’s Hospital of Fudan University, Shanghai, 201102 China; 6 Department of Gastroenterology, Peking Union Medical College Hospital, Peking Union Medical College & Chinese Academy of Medical Science, Beijing, 100730 China; 7 Department of Clinical Nutrition, Xinhua Hospital School of Medicine, Shanghai Jiao Tong University, Shanghai, 200092 China; 8 Department of Pediatric Gastroenterology, The First Hospital of Jilin University, Jilin, 130061 China; 9 Department of Gastroenterology, Beijing Children’s Hospital, Capital Medical University, Beijing, 100045 China; 10 Department of General Surgery, Sir Run Run Shaw Hospital, College of Medicine, Zhejiang University, Hangzhou, 310016 China; 11 Department of Gastroenterology, Shengjing Hospital of China Medical University, Shenyang, 110004 China; 12 Department of Pediatrics, West China Second Hospital, West China Women’s and Children’s Hospital, Chengdu, 610041 China; 13 Department of Pediatrics, Peking University Third Hospital, Beijing, 100191 China; 14 Department of Gastroenterology, Xi’an Children’s Hospital, Xi’an, 710003 China; 15 Department of Gastroenterology, Children’s Hospital of Zhengzhou University, Zhengzhou, 450053 China; 16 Department of Gastroenterology, Children’s Hospital of Nanjing Medical University, Nanjing, 210008 China; 17 Department of Gastroenterology, Shenzhen Children’s Hospital, Shenzhen, 518034 China; 18 Department of Pediatrics, Ruijing Hospital, School of Medicine, Shanghai Jiao Tong University, Shanghai, 350025 China; 19 Department of Gastroenterology, Children’s Hospital, Capital Institute of Pediatrics, Beijing, 100020 China

**Keywords:** Crohn’s disease, Pediatric inflammatory bowel disease, Quality indicators, Ulcerative colitis

## Abstract

**Background:**

Good quality of care for inflammatory bowel disease (IBD) depends on high-standard management and facility in the IBD center. Yet, there are no clear measures or criteria for evaluating pediatric IBD (PIBD) center in China. The aim of this study was to develop a comprehensive set of quality indicators (QIs) for evaluating PIBD center in China.

**Methods:**

A modified Delphi consensus-based approach was used to identify a set of QIs of structure, process, and outcomes for defining the criteria. The process included an exhaustive search using complementary approaches to identify potential QIs, and two web-based voting rounds to select the QIs defining the criteria for PIBD center.

**Results:**

A total of 101 QIs (35 structures, 48 processes and 18 outcomes) were included in this consensus. Structure QIs focused on the composition of multidisciplinary team, facilities and services that PIBD center should provide. Process QIs highlight core requirements in diagnosing, evaluating, treating PIBD, and disease follow-up. Outcome QIs mainly included criteria evaluating effectiveness of various interventions in PIBD centers.

**Conclusion:**

The present Delphi consensus developed a set of main QIs that may be useful for managing a PIBD center.

**Video Abstract**

**Supplementary Information:**

The online version contains supplementary material available at 10.1007/s12519-023-00691-0.

## Introduction

Inflammatory bowel disease (IBD) is a group of long-term conditions that inflame the gastrointestinal system. Ulcerative colitis (UC) and Crohn’s disease (CD) are the most common types. The accurate diagnosis of IBD should be based on comprehensive information from gastroenterology, imaging, pathology, esophagogastroduodenoscopy and ileocolonoscopy. Before diagnosing IBD, it is critical to rule out infections, rheumatic conditions, immunological deficiencies, and allergies. Consequently, cross-disciplinary collaboration is needed. Recurrent flares and disease remission characterize IBD progression, necessitating long-term therapy by IBD specialists and their teams to improve clinical results. In recent decades, a number of organizations, e.g., the European Crohn’s and Colitis Organization (ECCO), ImproveCareNow (ICN), the American Gastroenterological Association, and the Crohn’s Disease and Colitis Foundation of America (CCFA) have published different kinds of quality-control criteria for pediatric and adult IBD centers [1–5].

Two consensus and one criterion have been published by the Inflammatory Bowel Disease Group, Chinese Society of Gastroenterology, Chinese Medical Association, to assist adult gastroenterologists in setting up and managing standard IBD centers [6–8]. Since the phenotypes and management of pediatric IBD are not totally consistent with adult IBD, the consensus about adult IBD centers cannot be fully applied to children. Therefore, we formulated this consensus to offer quality control indicators for pediatric IBD based on national conditions in China.

## Methods

This consensus was formulated using the modified Delphi method. Using a formal group process, in which an expert panel discusses and iteratively evaluates the appropriateness of candidate quality indicators (QIs) using a two-round web-based survey.

### Development of quality indicators

An extensive search was performed in Medline, using multiple search strategies. QIs obtained from the documents retrieved in the literature search were collected and added to the initial comprehensive list of potential QIs. Existing clinical guidelines were reviewed to establish an ordered set of candidate QIs for subsequent evaluation. The Steering Committee (SC) included two pediatric gastroenterologists and one methodologist. During the preparation of the QIs, two pediatric gastroenterologists and one secretary evaluated the initial set of QIs.

### Selection of expert panel members and expert panel ratings

The expert panel members were selected by the Steering Committee. They were divided into two groups: the voting expert panel and the external audit expert panel. The voting expert panel members included 12 pediatric gastroenterologists, two adult gastroenterologists, one nutritionist, one surgeon, and one nurses. Members of the SC also participated in the voting process. Doctors and nurses were selected from different geographical locations in China. All of them were well-known experts in IBD and had published studies in the area of IBD in peer-reviewed journals. All participants also had preferential dedication to IBD and worked in either dedicated IBD clinics or IBD centers. The external expert panel members included four distinguished pediatric gastroenterologists and an adult IBD specialist.

A modified Delphi method was used to rate the appropriateness of each candidate QI. Before rating, the experts attended an online meeting and discussed each QI. They also worked on identifying additional QIs not included in the original list or modifying existing QIs that were judged to be imperfect. Redundant QIs were deleted, and new items were added according the panelists’ suggestions. In the first round, the experts rated each proposed QI individually without interaction with other members. Ratings were based on the review of an evidence report distributed to the panel in advance. After being analyzed, less significant QIs were deleted, and new ones were added following the experts’ suggestions. In the second round, the panelists were allowed to vote without adding or removing QIs. Voting was anonymous, and the votes of all panelists had the same weight in the analysis. The secretary finished the manuscript according to the second-round voting results and sent it to the external expert panel members for final review. The secretary made revisions according to the comments of external expert panelists.

### Rating system

The panelists rated the relevance of each candidate QI using a five-point scale: a. strongly agree; b. partially agree; c. agree; d. disagree, and e. strongly disagree. The strength of recommendations was classified into three levels according to the frequency of voting on different points. Level A (strongly recommended): the frequency of voting on point a is no less than 80%; level B (recommended): the frequency of voting on points a and b is no less than 80%; level C (suggested): the frequency of voting on points a, b and c is no less than 80%. QIs that did not achieve level C were deleted.

## Results

After the literature review, Seventy-two QIs were generated by literature review. After the first online meeting, 105 indicators were indentified. Duplicated QIs were deleted and one QI was added after two Delphi rounds. A final set of 101 QIs were included.

According to the Donabedian model [9], QIs were divided into three parts: structure, process and outcome. Structure refers to the basic structure of a pediatric IBD center, including the number of staff, medical conditions, facilities, etc. Thirty-five QIs were finally included in the “structure” part (Table 1). Level A indicators accounted for 57.1% (20/35). A multidisciplinary team (MDT) with a senior physician leader, two or more senior pediatric gastroenterologists, a decicated pathologist, radiologist, endoscopist was strongly recommended, as well as the technical support with endoscopy, infection detection, therapeutic drug monitoring and pre-treatment genotyping before thiopurine theray. The QIs also emphasize the importance of IBD registry, standard operation procedures, admission priority for emergency, and stransition approach to adult IBD centers while running a standard pediatric IBD center. Level B indicators accounted for 37.1% (13/35), and level C indicators accounted for 5.7% (2/35). Fifty-five to sixty percent of voting expert panel members were strongly agree with extentend MDT members e.g., dermatologist, rheumatologist, psychologist, geneticist, hematologist, and dedicated pharmacist.

**Table 1:** Quality-of-care items for the structure of pediatric inflammatory bowel disease centers

	Quality indicators	Strength of recommendation	Frequency of endorsement (%)				
			Strongly agree	Agree	Partially agree	Disagree	Strongly disagree
1	The center should have multidisciplinary team (MDT)	A	100	0	0	0	0
2	A multidisciplinary team for PIBD has:						
2.1	A dedicated PIBD senior physician as a leader	A	100	0	0	0	0
2.2	Two or more senior pediatric gastroenterologists whose subspecialty is IBD	A	95	5	0	0	0
2.3	A dedicated IBD nurse	B	65	25	10	0	0
2.4	A colorectal surgeon with experience in IBD surgery and clear referral pathway for complex IBD surgery	B	80	10	10	0	0
2.5	A dedicated pathologist	A	80	15	5	0	0
2.6	A dedicated radiologist	A	80	5	15	0	0
2.7	A dedicated dietitian/nutritionist	B	75	20	5	0	0
2.8	A dedicated endoscopist	A	85	10	0	5	0
2.9	Extended members of PIBD-MDT are suggested: dermatologist, rheumatologist, psychologist, geneticist and hematologist skilled in hematopoietic stem cell transplatation	C	60	10	30	0	0
2.10	A dedicated pharmacist	C	55	20	20	5	0
3	The center should have PIBD-MDT consultation system	A	90	5	5	0	0
4	PIBD-MDT platform is recommended	B	65	30	5	0	0
5	Facilities						
5.1	The center should have dedicated weekly PIBD clinic	B	65	20	10	5	0
5.2	The center should have sufficient inpatient beds for PIBD patients	B	50	30	20	0	0
5.3	The center is recommended to have access to capsule endoscopy or enteroscopy	B	65	20	15	0	0
5.4	The center should have at least two imaging techniques, such as magnetic resonance enterography, computed tomography enterography, and bowel ultrasound	B	65	25	5	5	0
5.5	The center should have access to esophagogastroduodenoscopy and colonoscopy	A	95	5	0	0	0
5.6	The center is recommended to have access to therapeutic endoscopic skills, such as balloon dilatations, endoscopic hemostasis	B	40	40	20	0	0
5.7	The center should have access to interferon-gamma release assays	A	80	10	10	0	0
5.8	The center should have access to *clostridium difficile* test	A	85	0	15	0	0
5.9	The center is recommended to have access to specific pathological techniques as acid-fast staining	A	90	5	5	0	0
5.10	The center is recommended to have access to immunohistochemistry to test cytomegalovirus infection	A	80	10	10	0	0
5.11	The center is recommended to have access to Epstein-Barr encoding region in situ hybridization	A	85	5	10	0	0
5.12	The center is recommended to have access to immunohistochemistry to test HBV infection	B	75	5	15	5	0
5.13	The center is recommended to have access to measuring through level and antibodies of anti-TNF biologics	A	80	5	15	0	0
5.14	The center is recommended to have access to TPMT and NUDT15 genotypes	A	55	40	5	0	0
6	The center has a PIBD registry	A	80	10	10	0	0
7	The center has standard operation procedures for PIBD management	A	80	15	0	5	0
8	The center should have PIBD checklist system	A	85	5	5	5	0
9	Patient support:						
9.1	The center has 24-h contact-line or access, for PIBD patients	B	45	35	20	0	0
9.2	The center has online educational information on PIBD	B	60	35	5	0	0
9.3	The center is recommended to have regular meetings between patients and IBD staff to provide educational information	B	75	5	20	0	0
9.4	The center provides priority admission for IBD children who are in emergency or critically ill	A	85	10	5	0	0
9.5	The center has access to transit patients to adult IBD centers	A	85	10	5	0	0

*PIBD* pediatric inflammatory bowel disease, *HBV* hepatitis B virus, *TNF* tumor necrosis factor, *TPMT* thiopurine S-methyltransferase, *NUDT15* nucleoside diphosphate liked to moiety X-type 15, *IBD* inflammatory bowel disease, *MDT* multidisciplinary team

“Process” refers to the process of managing IBD that is needed in pediatric IBD centers. In the “process” section, 48 QIs were included (Table 2). Among them, 77.1% (37/48) were recommended as level A. All voting experts strongly agreed with ruling out intestinal infections before UC was diagnosed. A complete diagnostic classification and full communication with children and family were also considered to be an essential part in the process of managing IBD. 18.8% (9/48) QIs were recommended as level B, and 4.2% (2/48) were recommended as level C. QI that suggest genetic test in children with disease onset before two years obtained 60% vote of “Strongly agree”. Quality-of-life assessment at diagnosis gained only half vote of “Strongly agree”.

**Table 2:** Quality-of-care items for the process of pediatric inflammatory bowel disease center

	Quality indicators	Strength of recommendation	Frequency of endorsement (%)				
			Strongly agree	Agree	Partially agree	Disagree	Strongly disagree
1	Diagnosis and differential diagnosis						
1.1	Stool routine test and stool culture should be done before diagnosis of UC is made	A	100	0	0	0	0
1.2	Clostridium difficile should be ruled out before diagnosis of IBD is made	A	80	10	5	5	0
1.3	Tuberculosis infection is routinely ruled out before diagnosis of IBD is made. Diagnostic therapy is performed in patients who are suspicious with tuberculosis	A	85	5	5	5	0
1.4	Examination and evaluation of the whole gastrointestinal tract should be done when diagnosing IBD	B	70	20	0	10	0
1.5	Genetic test is advised for children whose symptoms start before age 2	C	60	15	25	0	0
1.6	Genetic test is advised for children who have family history of IBD	B	75	10	15	0	0
1.7	Genetic test is advised for children who are refractory to routine IBD treatment	B	75	10	10	5	0
2	Evaluation of IBD						
2.1	A complete diagnostic classification, including disease phenotype, extent, severity, behavior, perianal disease, nutrition and growth status	A	100	0	0	0	0
2.2	Malnutrition should be screened at the time of diagnosis	A	80	15	5	0	0
2.3	Nutrition status should be evaluated at the time of diagnosis	A	90	10	0	0	0
2.4	Bone age should be checked when patients have growth retardation	B	65	30	5	0	0
2.5	Scores should be used to evaluate severity of IBD endoscopic appearance	A	85	5	10	0	0
2.6	Ileum should be included during colonoscopy, except for patients with colonic stenosis	A	90	5	5	0	0
2.7	Multiple biopsies (2 or more per segment) should be taken from all segmengts of gastrointestinal tract during colonoscopy	A	85	15	0	0	0
2.8	Enteroscopy, capsule endoscopy or imaging techniques (magnetic resonance enterography or computed tomography enterography) can be used to assess small intestinal disease	A	95	0	5	0	0
2.9	When assessing small intestinal disease, capsule endoscopy or enteroscopy is prior to imaging techniques (magnetic resonance enterography or computed tomography enterography)	B	55	30	5	5	5
2.10	Assessing quality of life is advised at the time of diagnosis	C	50	20	30	0	0
2.11	Nutrients levels should be monitored for patients who have nutrients deficiency	B	70	20	10	0	0
2.12	Bone metabolism and bone mineral density should be tested in children who have moderate to severe malnutrition, or receive corticosteroid treatment for more than 3 mon	A	85	10	10	0	0
3	Treatment						
3.1	Fully communication with children and their family about IBD and therapeutic strategy is important, to achieve their cooperation	A	100	0	0	0	0
3.2	Informed consent should be signed before using off-lable medications	A	95	5	0	0	0
3.3	If it is possible, age-appropriate vaccination should be finished before starting immunomodulators	B	65	30	0	5	0
3.4	HBV infection should be excluded before starting immunomodulators	A	90	10	0	0	0
3.5	Tuberculosis screening, including chest X-ray/chest CT, tuberculin skin test and interferon-gamma release assays, should be performed before starting immunomodulatory	A	85	15	0	0	0
3.6	Immediate admission and enough intravenous corticosteroids is advised for ASUC children	A	85	10	0	5	0
3.7	For patients with ASUC who have no response to enough intravenous corticosteroid, second-line therapy (cyclosporine, tacrolimus, anti-TNFs, or surgery) is advised in 7 d	B	75	20	0	5	0
3.8	Thiopurines, methotrexate or anti-TNFs are advised for children who need more than 2 cycles of corticosteroid	A	80	20	0	0	0
3.9	Routine therapeutic drug monitoring(reactive or proactive) is used to optimize therapies	A	85	5	5	5	0
3.10	Regular blood test should be taken to monitor adverse effects during thiopurine or methotrexate treatment	A	95	5	0	0	0
3.11	6-TGN and 6-MMP is advised to optimize/monitor thiopurine therapy	B	70	15	10	5	0
3.12	Folic acid is supplied during methotrexate treatment	A	80	10	5	5	0
3.13	Nutritional therapy should be done according to the results of nutritional assessment	A	100	0	0	0	0
3.14	Nutrients should be supplied for children who have nutrients deficiency at the time of diagnosis	A	100	0	0	0	0
3.15	Surgical risk assessment and perioperative management should be performed for children undergoing elective surgery	A	95	0	5	0	0
4	Follow-up						
4.1	Routine follow-up is recommended after ileocolonic intestinal resection in children with CD	A	95	5	0	0	0
4.2	Ileocolonoscopy should be performed 6–9 months after ileocolonic anastomosis	A	90	10	0	0	0
4.3	Prophylactic treatment is suggested after ileocolonic intestinal resection in CD patients with high-risk factors^a^	A	80	15	5	0	0
4.4	Clorstium difficile infection should be ruled out during the flare-up	A	80	10	5	5	0
4.5	Cytomegalovirus infection should be ruled out during flare-up	A	80	5	10	5	0
4.6	Epstein-Barr virus infection should be ruled out during flare-up	A	80	5	10	5	0
4.7	Biomarkers(such as fecal calprotectine), colonoscopy, and/or radiology techniques is used to assess extent, severity and complications of disease during flare-up	A	85	10	5	0	0
4.8	Height and weight is recorded and plotted on each clinical visiting	A	80	15	5	0	0
4.9	Nutritional status and nutrients (such as, iron, vitamin D, vitamin B12, folic acid) should be assessed during and after a flare	A	85	10	5	0	0
4.10	Nutritional status and nutrients (such as, iron, vitamin D, vitamin B12, folic acid) should be assessed once a year for patients in remission	A	80	10	10	0	0
4.11	Endoscopic follow-up should be done every 1–2 y for patients in remission	A	90	5	5	0	0
4.12	Severity of disease should be regularly monitored for patients in remission	A	95	5	0	0	0
4.13	Fecal calprotectin should be regularly monitored for patients in remission	A	80	10	10	0	0
4.14	Live vaccine is not advised during immunomodulator therapy	A	95	0	5	0	0

*UC* ulcerative colitis, *HBV *hepatitis B virus, *CT* computerized tomography, *ASUC* acute severe ulcerative colitis, *6-TGN* 6-thioguanine nucleotide, *6-MMP* 6-methylmercaptopurine, *CD* Crohn’s disease, *IBD* inflammatory bowel disease

^a^High-risk factors: extensive disease, early surgery, repeat surgery, extensive small bowel resection (> 50 cm), bowel perforation or stricture, perianal disease, smoking

“Outcome” refers to the clinical outcomes and prognosis of children with IBD after various interventions by health care providers in IBD centers. A total of 18 QIs were included in this part (Table 3). Of these indicators, 33.3% (6/18) were A-level indicators, and 66.7% (12/18) were B-level indicators.

**Table 3:** Quality-of-care items for outcomes of pediatric inflammatory bowel disease centers

	Quality indicators	Strength of recommendation	Frequency of endorsement (%)				
			Strongly agree	Agree	Partially agree	Disagree	Strongly disagree
1	The time interval between reservation of hospitalization and admission	B	70	15	15	0	0
2	Proportion of patients in remission	B	75	15	5	5	0
3	Proportion of patients who are in steroid-free remission^a^	B	75	10	15	0	0
4	Proportion of patients who are steroid-free^a^	B	65	25	10	0	0
5	Proportion of patients who are receiving exclusive enteral nutrition^a^	A	80	5	15	0	0
6	Proportion of patients who have perianal remission^a^	A	80	5	10	5	0
7	Mean length of hospitalization per year^a^	B	65	15	20	0	0
8	Annual rate of unplanned emergency visits	A	80	15	5	0	0
9	Proportion of patients who are at risk of malnutrition^a^	B	70	15	15	0	0
10	Proportion of patients who are malnorished^a^	B	75	10	15	0	0
11	Proportion of patients who have growth retardation^a^	B	75	10	15	0	0
12	Proportion of patients who achieved nutritional improvement^b^	A	80	15	5	0	0
13	Proportion of ASUC patients who have surgery^a^	A	80	5	10	5	0
14	Fatality rate of ASUC^a^	A	80	10	5	0	5
15	Complication rate of surgery (emergency surgery or elective surgery)	B	70	20	5	5	0
16	Death rate of surgery (emergency surgery or elective surgery)	B	70	20	5	0	5
17	Number of unplanned re-surgery cases after bowel/intestinal resection	B	65	20	15	0	0
18	Proportion of patients with normal quality of life	B	60	20	15	5	0

*ASUC* acute severe ulcerative colitis

^a^Excluding the patients diagnosed in the last 4 months

^b^The change in nutritional status between baseline and 4 months after treatment

## Discussion

High-quality care for children with IBD is critical in reducing complications and disability rates and maintaining long-term remission. Compared with adults, early standardized and structured management is more conducive to long-term clinical prognosis in children due to early onset disease.

The management of IBD often requires multidisciplinary cooperation, so an IBD center needs to established in  a hospital with a multidisciplinary expert team [10–12]. The MDT consists of a core team and an extended team [10, 12–14]. The IBD core team is mainly composed of pediatric IBD specialists, pediatric gastroenterologists or gastrointestinal surgeons with clinical experiences in IBD, specialized endoscopists and IBD nurses. Since IBD requires long-term monitoring and management, the experience of general doctors is insufficient to adequately manage IBD patients. Therefore, pediatricians with experiences on IBD management are important in the IBD core team. Registered  IBD nurses should also be included in the IBD core team. They can communicate with doctors about disease information; on the other hand, they can provide children and their families with adequate training, psychological support, and consultation of IBD-related information [15–17]. This work can not only increase the patients’ compliance to the treatment but also brings better clinical outcomes. In this consensus, more than 85% of the experts voted to agree or strongly agree on the importance of the MDT core team. However, only 70%–75% of the experts agreed or strongly agreed on the importance of extended teams in IBD centers. In the position paper developed by ECCO in 2020, 96%–100% of experts considered it important to have an extended IBD-MDT team (including psychologists, rheumatologists, stoma management specialists, pharmacists, dermatologists, infection specialists, etc.) [2]. The differences in the views of Chinese and Western pediatric experts on the IBD-MDT extended team may be related to the different medical system in these two regions. In China, high-quality medical resources are mostly concentrated in large cities. Therefore, some hospitals in areas or regions with low social-economical level have not established relevant pediatric subspecialties; or there are no relevant subspecialties established in the region where that hospital is located. On the other hand, it might be explained by the underestimation of the importance of the impact of IBD as a chronic disease on multiple systems.

The PIBD-MDT platform and PIBD-MDT consultation system provide guarantees for the smooth operation of MDTs. The PIBD-MDT platform should have a fixed place and provide a multimedia network system. Therefore, MDT members can easily have access to electric medical records, imaging and endoscopic images during discussion. Regular MDT meetings can be held based on this platform [11]. The PIBD-MDT consultation system should state the responsibilities of team members, the application process of consultations, and the location and time of consultations [11]. MDT members should update their knowledge according to the latest guidelines. Therefore, it is recommended that the PIBD-MDT hold regular meetings to discuss intractable cases and learn the latest literature. Team members are required to participate in IBD-related academic activities and be able to conduct IBD-related research [11, 14, 18].

IBD centers should facilitate the process of diagnosis and treatment for children with IBD. To date, it is still difficult for children with IBD to see a physician specialized in because of the low number of pediatricians specialized in IBD in China. Some patients who live in remote rural areas might stop taking their medications without the guidance of medical care due to inconvenient traffic or economic issues, which results in a poor prognosis. Studies have demonstrated that the good structure of the center, including an identified IBD clinic, dedicated nurses, and early access to IBD specialists, can lead to better clinical outcomes [19, 20]. IBD centers should have appropriate supporting facilities and technologies to ensure the operation of IBD management. These include an identified IBD clinic, relatively fixed IBD beds, imaging technologies (magnetic resonance enerography, CT enterography, bowel ultrasound, etc.) [21], access to specific laboratory techniques (γ-interferon release test, Clostridium difficile tioxin, etc.), therapeutic endoscopic skills, specific pathological techniques (staining for acid fast bacilli for tuberculosis, immunohistochemistry, in situ hybridization, etc.), etc. [1, 22, 23]. In this consensus, only 40% of the voting experts strongly agreed that the center has access to therapeutic endoscopic skills. This result might be due to the lack of well-developed therapeutic endoscopic skills in pediatric endoscopists, as well as the lower incidence of complications in IBD children than in the adult cohort.

Adequate patient education and services affect medication adherence and disease prognosis. IBD centers can provide patient education and support in various formats, including online and offline activities, patient educational courses, disease knowledge brochures. Medical staff in the IBD centers can provide remote counseling services in cases of disease recurrence or emergency through a hotline or a social media platform. In this consensus, only 45% of the experts strongly agreed with the establishment of a 24-hour consultation route (e.g., telephone, a social media platform, etc.), and 60% strongly agreed with the establishment of an online mission system. The reason might be the difficulty in reaching IBD physicians or nurses at any time due to the lower ratio of medical and nursing staff per patient compared with Western countries. As a result, patient support is relatively weak in China. These factors can influence the decision of specialists when voting.

With regard to the diagnosis and differential diagnosis of IBD in children, experts differ in their opinions regarding the timing and indications of genetic testing. Seventy-five percent of experts strongly agreed with genetic testing for children who were refractory or had a family history of IBD, and 60% strongly agreed with routine genetic testing for children with early symptom onset (under two years of age). There may be multiple reasons for these outcomes. First, the cost of genetic testing is high and not covered by health insurance. It is not affordable for some families. In addition, rare data concerning the prevalence of gene deficiency in refractory IBD or infant IBD can be obtained to support experts in making appropriate decisions for voting. Last but not least, genetic testing can only guide the treatment of a small number of children.

For the assessment of IBD, there are three main indicators with which experts agree less strongly. First, 65% of experts strongly agree with testing bone age in children with growth retardation. Some experts consider that growth retardation in IBD children can be corrected after disease control and nutritional therapy. Bone age testing is not an immediate need for these children. Therefore, this item is not necessarily used as a main indicator to assess a PIBD center. Second, only 55% of experts strongly agreed that enteroscopy (including capsule endoscopy or double-balloon enteroscopy) is preferable to imaging for the evaluation of small bowel lesions. In 2020, the ECCO position paper showed that 94% of voting experts considered enteroscopy to be desirable rather than essential when imaging techniques are doubtful or negative in the presence of a strong clinical suspicion of CD [2]. Enteroscopy and imaging techniques have different emphases for CD. Enteroscopy focuses on mucosal lesions, whereas imaging techniques focus on lesions in the intestinal wall and outside the lumen. For those who have stenosis or bowel obstruction, imaging techniques are more preferable. Third, 50% of the experts strongly agreed with the assessment of quality of life in children with IBD. To date, there are no suitable scales for evaluating the quality of life of children with IBD in China. Therefore, the development of a valid quality of life scale is an urgent need.

In terms of the treatment of PIBD, the proportion of strong agreement (65% and 70%, respectively) was lower for the items of vaccination and monitoring metabolites during thiopurine therapy than for the rest of the items. Infectious diseases are predominant in pediatric disorders. Children with IBD are more susceptible to infections due to poor nutritional status and the use of immunosuppressive medications. Although the effectiveness of vaccination may be weakened under immunosuppressive conditions, vaccination is recommended in children with IBD [24–26]. The results of the voting reflect the current views of domestic IBD specialists on vaccination.

The indicators in the outcome assessment are closely related to the quality of disease management and prognosis. The assessment of outcomes can reflect the aspects of the quality of care that need to be improved. In this consensus, the proportion of level-A recommendations was 33.3%, and the remaining indicators were all graded as level-B. The proportion of strongly agree on each item did not exceed 80%. These voting results indicate that more efforts need to be made by health care providers on suitable indicators of outcome assessment to accurately evaluate the quality of care in IBD centers.

Our study has several limitations. Firstly, although there were two-round multidisciplinary expert panel discussion, selection bias exist. The members in the Steering Committee who selected the initial QIs were not multidisciplinary. Secondly, patients were not included as panel members in this project. So the consensus focuses more on standards of supervising a PIBD center, rather than PIBD care, especially on patients’ perspective. Furthermore, this consensus reflects the local situation in China with its own limitation. Generalization of the results might not reflect the ideal care that should be provided to patients with PIBD.

In conclusion, this consensus built primary criteria of running a PIBD center in China. Due to current medical situation and health systems in China, the consensus might not be generalized worldwide. The results might require adaptation according to local conditions. Further revision and updating of the consensus should be done according to the increasing evidences from Chinese investigators in the near future.

## Data Availability

The datasets generated and/or analyzed during the current study are available from the corresponding author on reasonable request.
